# First-principles study of nonmetal doped monolayer MoSe_2_ for tunable electronic and photocatalytic properties

**DOI:** 10.1038/s41598-017-17423-w

**Published:** 2017-12-06

**Authors:** Yafei Zhao, Wei Wang, Can Li, Liang He

**Affiliations:** 1 National Laboratory of Solid State Microstructures, School of Electronic Science and Engineering and Collaborative Innovation Center of Advanced Microstructures, Nanjing University, Nanjing, 210093 China; 2 Center for Coordination Bond Engineering, College of Materials Science and Engineering, China Jiliang University, Hangzhou, 310018 China

## Abstract

Recently, two dimensional transition metal dichalcogenides become popular research topics because of their unique crystal and electronic structure. In this work, the geometrical structure, electronic, electrical transport, redox potentials and photocatalytic properties of nonmetal (H, B, C, Si, N, P, As, O, S, Te, F, Cl, Br and I) doped monolayer MoSe_2_ were investigated by first principle calculations. The binding energy indicates that nonmetal doped MoSe_2_ are energetically favorable compared to Se vacancies, except B- and C-doped. We have found that nonmetal dopants with an even number of valence electrons doped MoSe_2_ have p-type conductivity. On the contrary, nonmetal dopants with an odd number of valence electrons doped MoSe_2_ have p-type or n-type conductivity; and they have better photocatalytic performance.

## Introduction

Transition metal dichalcogenides (TMDCs) have unique structural and electronic properties. Consequently, they possess various conducting properties that include insulating, semiconducting, conducting and even superconducting. And these cause potential applications in electronics, spintronics and optoelectronics^1–8^. TMDCs family has more than 40 compounds (including MoS_2_, WS_2_, MoSe_2_, WSe_2_ and WTe_2_ *etc*) with the formula of MX_2_^9,10^. These TMDCs materials have been obtained by micromechanical exfoliation or liquid exfoliation (due to the weak van der Waals bonding interactions in the adjacent sandwiched layers)^11–13^, chemical vapor deposition (CVD)^14^ and molecular beam epitaxy (MBE)^15,16^. However, vacancies inevitably occur in the growth process of TMDCs^17–19^.

The introduction of dopant atoms into the vacancies is considered as a promising method of modulating their electronic, magnetic and transport properties of monolayer TMDCs^20–29^. Experimentally, Zn- and Co-doped MoS_2_ nanosheets have been realized and demonstrate potential applications in the field of electronics, optoelectronics, spintronics and photocatalysis^30,31^. Moreover, a series of work have reported that ultrathin N- and P-doped MoS_2_ nanosheets have demonstrated enhanced hydrogen evolution reaction (HER) catalysis, enhanced oxygen reduction reaction (ORR) and efficient degradation of methyl orange and RhB, respectively^32–36^. This is due to they can promote the charge transfer in the photocatalytic reaction. These results suggest that doping nonmetal (NM) atoms is an effective way to promote the catalytic performance.

However, the above articles lack the calculations on the electrical transport of NM doped systems. Moreover, systematically understanding the modification on the physical and chemical properties of the NM doped monolayer MoSe_2_ is necessary. In this work, we provide a comprehensive investigation on the geometrical structure, binding energy, electronic, optical and photocatalytic properties of substitutionally doped monolayer MoSe_2_ with a series of NM atoms, such as H, B, C, Si, N, P, As, O, S, Te, F, Cl, Br and I, by employing first-principle calculations. And we found the parity of the valence electrons of the dopants causes a dramatic difference on electrical transport, molecular state, redox potentials and photocatalytic activity.

## Computational Methods

**Figure 1:**
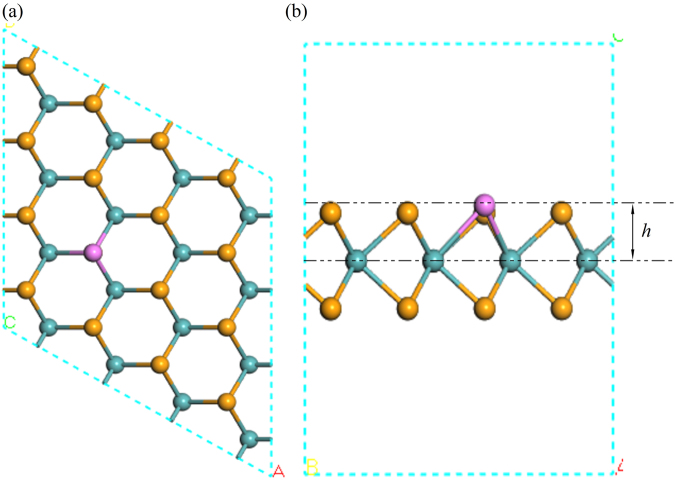
(**a**) Top view and (**b**) side view of the crystal structure of a 4 × 4 × 1 doped supercell monolayer MoSe_2_. The blue, yellow and purple spheres denote the Mo, Se and NM atoms, respectively.

The geometrical structure, electronic and optical properties of undoped and NM doped monolayer MoSe_2_ are calculated within the framework of density functional theory by using the CASTEP package. Norm-conserving pseudopotentials and Perdew-Burke-Ernzerhof (PBE) function of the generalized gradient approximation (GGA) are used for the electron-ion interactions and exchange-correlation potential, respectively^37,38^. The adopted monolayer structure is a 4 × 4 × 1 supercell containing 16 Mo (blue ball), 31 Se (yellow ball) and 1 NM atoms (purple ball) (Fig. 1(a)). The high cutoff energy for the plane-wave basis is set at 750 eV and the Brillouin zone is sampled by a 9 × 9 × 1 *k*-point sampling grid. A vacuum layer of 15 Å is adopted in the direction perpendicular to the monolayer surface to avoid the interactions between periodic slabs. The structural optimization is continued until the convergence tolerance of energy, maximum force and maximum displacement are less than 5.0 × 10^−6^ eV/atom, 0.01 eV/Å and 1.0 × 10^−3^ Å, respectively.

We have calculated the binding energies (*E*_b_) of all the doped systems to assess the stability of doped MoSe_2_:

1 $${E}_{{\rm{b}}}={E}_{{\rm{vacany}}}+{\mu }_{{\rm{NM}}}\,-\,{E}_{{\rm{doped}}}$$

where *E*_doped_ is the total energy of the monolayer MoSe_2_ with one Se atom replaced by the NM atom, *E*_vacany_ is the total energy of the monolayer MoSe_2_ with one Se vacancy, and *μ*_NM_ is the chemical potential of the dopant and is calculated with respect to the elemental bulk or gas in nature. Positive *E*_b_ suggests that doped atom is energetically favorable to sit at the substitutional site of Se.

## Results and Discussions

### Binding energies and optimized Structures

Table 1 lists the calculated results of the doped systems. All of them (except for B- and C-doped) have positive *E*_b_, indicating that their formations are exothermic reaction and therefore stable. The *E*_b_ of the VIA (O, S and Te) group doped is the largest (>2 eV). This is due to the dopants have the same valence electron structure as Se. Thus they are most stable when fill up the Se vacancy. O-doped system, overall, has the largest *E*_b_ (3.78 eV), since O atom has the highest electronegativity. The *E*_b_ of the VA (N, P and As) and VIIA (F, Cl, Br and I) group doped are smaller (~1 eV). This is probably due to the absent or extra one electron cost some formation energy. Doping of NM atoms with the IA (H), IIIA (B) and IVA (C and Si) groups is less energetically favorable, with *E*_b_ of ~0 eV. This can be attributed to their valence electron structures have the largest difference compared with Se.

**Table 1:** The equilibrium supercell lattice parameters *a* with unit Å. *d*_Mo-NM_ is the bond lengths of Mo-NM bonds with unit Å. *h* is the sheet thicknesses from NM atom to the reference Mo atom plane with unit Å. *E*_b_, *E*_g_ *E*_F_, *E*_CBM_ and *E*_VBM_ are the calculated binding energies, band gap, Fermi level, the energy edge of CBM and VBM relative to vacuum level, respectively, with unit eV.

Dopant	*a*	*d*_Mo-NM_	*h*	*E*_b_	*E*_F_	*E*_VBM_	*E*_CBM_	*E*_g_
H	3.332	2.054	0.888	0.13	−3.00	−3.95	−2.29	1.66
B	3.350	2.145	0.801	−0.85	−3.75	−4.00	−2.35	1.65
C	3.344	2.041	0.795	−0.81	−3.38	−3.38	−1.82	1.56
Si	3.364	2.415	1.181	0.13	−3.30	−3.30	−1.84	1.46
N	3.337	2.018	0.927	0.46	−3.54	−3.91	−2.35	1.56
P	3.348	2.446	1.531	1.19	−3.80	−3.80	−2.26	1.54
As	3.349	2.595	1.749	0.92	−3.86	−3.86	−2.30	1.56
O	3.332	2.088	1.047	3.78	−3.40	−3.40	−1.81	1.63
S	3.346	2.436	1.522	2.67	−3.39	−3.39	−1.76	1.61
undoped	3.351	2.570	1.641	—	−3.36	−3.36	−1.74	1.62
Te	3.358	2.762	1.932	2.04	−3.33	−3.33	−1.73	1.60
F	3.341	2.276	1.253	0.34	−2.52	−3.94	−2.28	1.66
Cl	3.353	2.534	1.621	1.31	−2.43	−3.92	−2.43	1.51
Br	3.358	2.673	1.803	1.04	−2.45	−3.90	−2.27	1.63
I	3.363	2.853	2.037	0.94	−2.48	−3.87	−2.26	1.61

Although their *E*_b_ varies a lot, their lattice structures stay constant, within 0.6%. On the other hand, the local structure and the electronic properties have been modified dramatically. The Mo-NM bond lengths (*d*_Mo-NM_) and *h* (the sheet thicknesses from NM atom to the reference Mo atom plane, Fig. 1(b)) of doped MoSe_2_ vary as much as 21.5% and 51.6%, respectively. This is mostly due to the variation of the atomic radius and electronegativity of NM atoms. More details will be discussed later.

### Electronic and electrical transport properties

**Figure 2:**
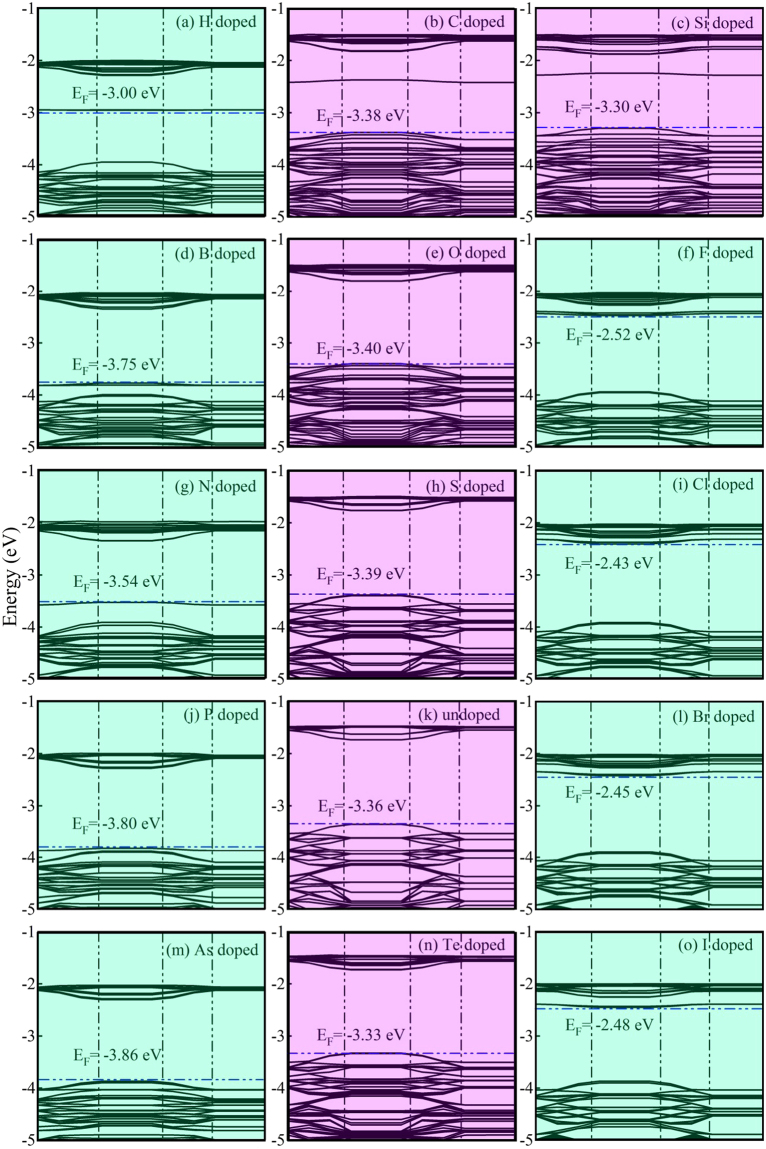
The band structures of undoped and NM doped monolayer MoSe_2_. The light blue and light purple background colors are represent the band structures of the odd and even number valence electrons doped systems, respectively. NM dopants with an even number of valence electrons are generally p-type conductivity. On the contrary, NM dopants with an odd number of valence electrons can provide effective p-type or n-type conductivity.

**Figure 3:**
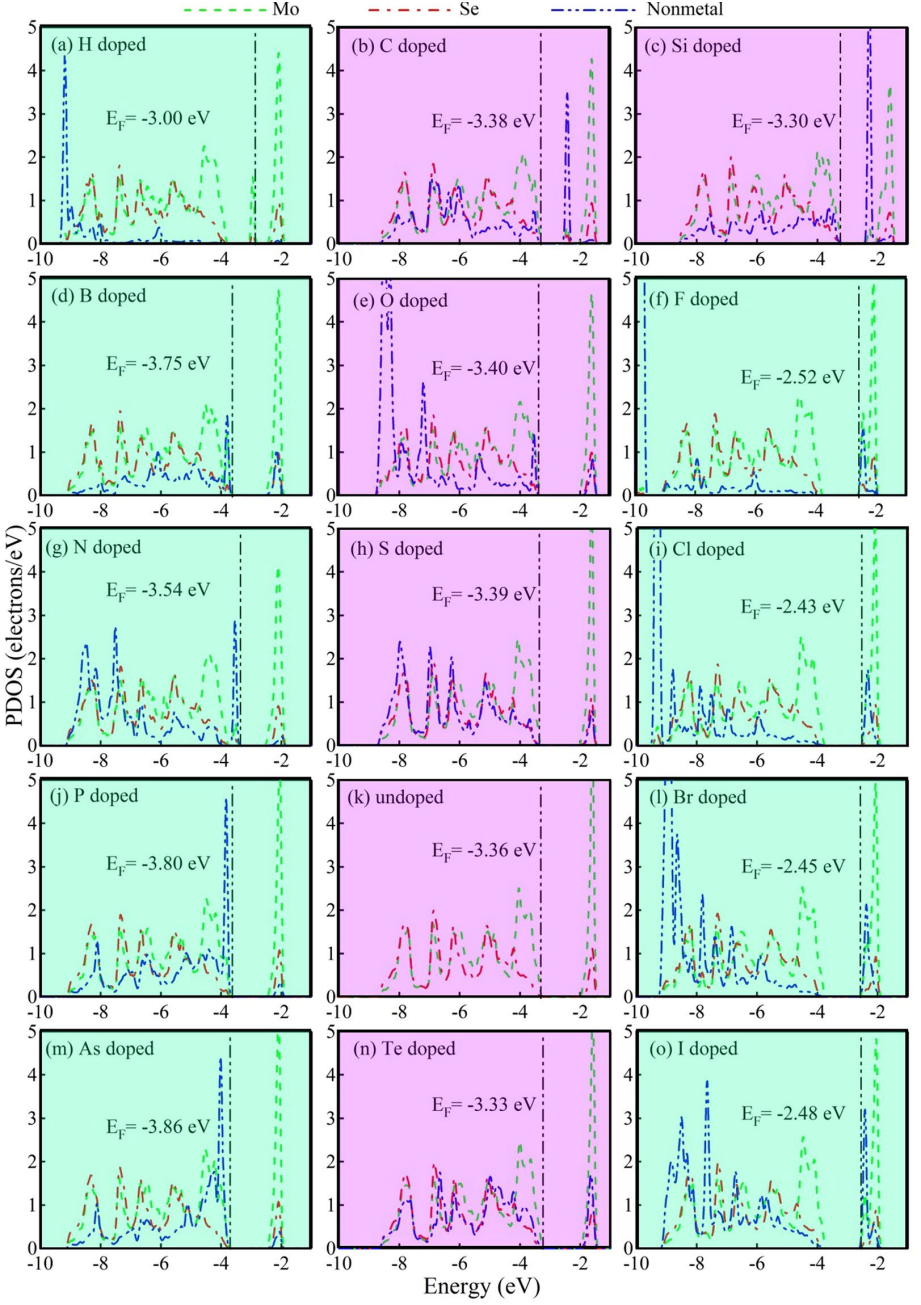
The average PDOS of Mo, Se and NM atoms of undoped and NM doped monolayer MoSe_2_.

To explain how doped NM atoms modify the electronic properties of monolayer MoSe_2_, we have calculated the band structures and partial density of states (PDOS) of individual Mo, Se and NM atoms of all the systems, as shown in Figs 2 and 3, respectively. For undoped monolayer MoSe_2_, conduction band (CB) and valence band (VB) are composed of Mo-4*d* and Se-4*p* states, and each Mo atom can provide four electrons interacting with the surrounding six Se atoms. For IVA (C and Si) and VIA (O, S and Te) group doped systems (NM dopants with even number of valence electrons), the Fermi level (*E*_F_) of doped MoSe_2_ is almost unchanged (−0.04 ~ 0.06 eV, see Table 1) compared to the undoped one and is slightly above the valence band maximum (VBM), seen in Fig. 2. These phenomena are consistent with the results in C-, Si-, O-, Se- and Te-doped monolayer MoS_2_ or WSe_2_^21,28,29^. Thus, NM dopants with an even number of valence electrons doped monolayer MoSe_2_ is still generally a p-type semiconductor.

For IIIA (B) and VA (N, P and As) group doped cases, the *E*_F_ lowered about 0.18~0.50 eV compared to the undoped one. The similar results have also been reported in B- N-, P- and As-doped monolayer MoS_2_ or WSe_2_ and N-, P- and As-doped monolayer MoSe_2_^21,28,39,40^. Thus, they are still a p-type semiconductor. For IA (H) group doped case, a half-filled impurity level (mostly composed of Mo-4*d* states) appears within the band gap and lifts the *E*_F_ into the middle of the band gap. Thus p-type conductivity is suppressed. For VIIA (F, Cl, Br and I) group doped systems, an additional valence electron results in the donor impurity level (consisting Mo-4*d* and NM-*p* states) and the *E*_F_ is lifted close to the conduction band minimum (CBM). Thus, they become an n-type semiconductor, consistent with the reported results^21,28,39,40^. Thus, we can conclude that NM dopants with an odd number of valence electrons doped MoSe_2_ can have p-type or n-type conductivity. On the contrary, NM dopants with an even number of valence electrons can only provide p-type conductivity. This suggests a possible way to realize bipolar electrical transport properties by tuning the dopants, which is essential for practical applications in optoelectronics and electronics.

**Figure 4:**
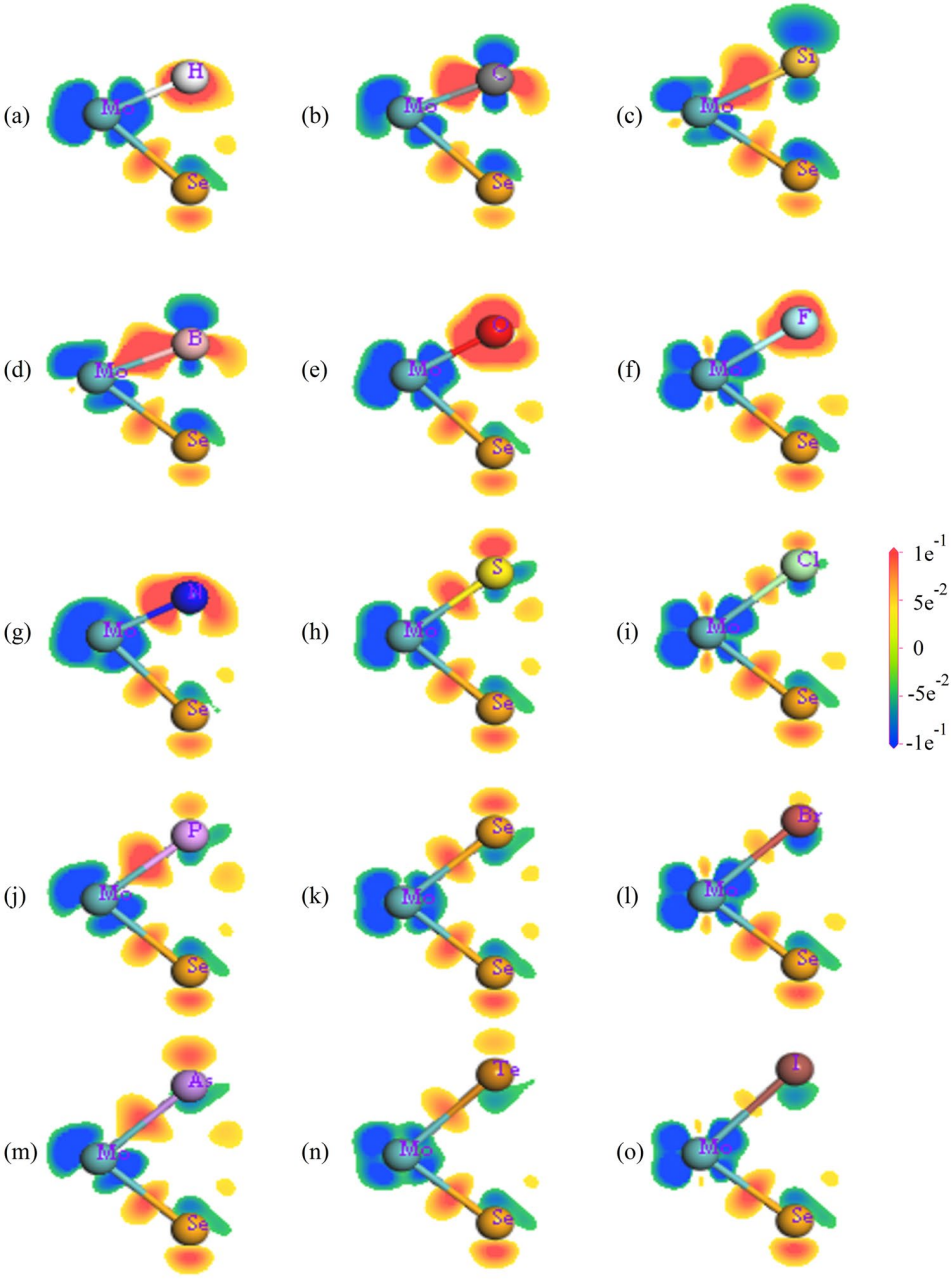
The electron density difference of undoped and NM doped monolayer MoSe_2_. Mo-4*d* and Se-4*p* states (green and red lines) are basically invariable, while the NM-*p* (or -*s*) states (blue lines) demonstrate huge difference. Thus, the dopants can only affect the local electronic structure.

We further observe the PDOS of all doped systems found that Mo-4*d* (green lines) and Se-4*p* states (red lines) are basically invariable, while the NM-*p* (or -*s*) states (blue lines) demonstrate huge difference (see Fig. 3). Therefore, we believe that the dopants can only affect the local electronic structure. This is also verified by the calculated electronic density difference. Overall, the dopants only affect the electronic distribution of the three nearest neighboring Mo atoms, other atoms are unaffected. Thus, in Fig. 4, we only demonstrate the electronic density difference of the NM and the closest Mo and Se atoms. The blue (or red) regions indicate the electron loss (or accumulation), and the red regions between the two atoms indicate that they form a covalent bond. Thus, the Se and the Mo atom are ionized; meanwhile, a covalent bond is formed between them in undoped monolayer MoSe_2_. It is found that Mo-NM ionic bonds are formed in the N-, O- and F-doped system, due to their large electronegativity. On the other hand, covalent bonds with different strength are formed for other dopants. Thus we believe the change of the globe electronic properties of the doped MoSe_2_ is due to this local change of electron distribution.

### Optical and photocatalytic properties

We have also calculated the optical properties of all the systems and the results show that NM dopants slightly affect the optical absorption edge and optical absorption areas in the range of 300 to 900 nm (see Supplementary Fig. S1). On the other hand, this work also provides the highest occupied molecular state (HOMO) and lowest unoccupied molecular state (LOMO) of all the systems (show in Fig. S2). We have found that the physical location of HOMO and LUMO can be tuned by different NM dopants^28,41^.

For undoped MoSe_2_, the HOMO and LUMO are made up of Mo-4*d* states, thus they are located at all the Mo atoms simultaneously (Fig. S2(k)). And the photogenerated electrons (e^−^) and holes (h^+^) will be easily recombined on Mo atoms, which reduce the photocatalytic efficiency. For IVA (C and Si) group doped MoSe_2_, both the HOMO and LUMO mainly locate at the NM atom and its three nearest Mo atoms. For VIA (O, S and Te) group doped MoSe_2_, similar to undoped MoSe_2_, both the HOMO and LUMO mainly locate at the Mo atoms across the film. Thus, in these two cases, the photogenerated electrons e^−^/h^+^ have a good chance to recombine.

For IA (H) group and VIIA (F, Cl, Br and I) group doped MoSe_2_, the distribution of HOMO states is the same as the undoped MoSe_2_, while the LUMO states are mainly located at the NM atom and its three nearest Mo atoms. For IIIA (B) doped MoSe_2_, the distribution is opposite to them. For VA (N, P and As) group doped MoSe_2_, the HOMO states locate at around NM atom and its surrounding Mo atoms, and the LUMO states locate at Mo atoms further away from NM atoms. In these three cases, the HOMO and LUMO states are separated in real space.

**Figure 5:**
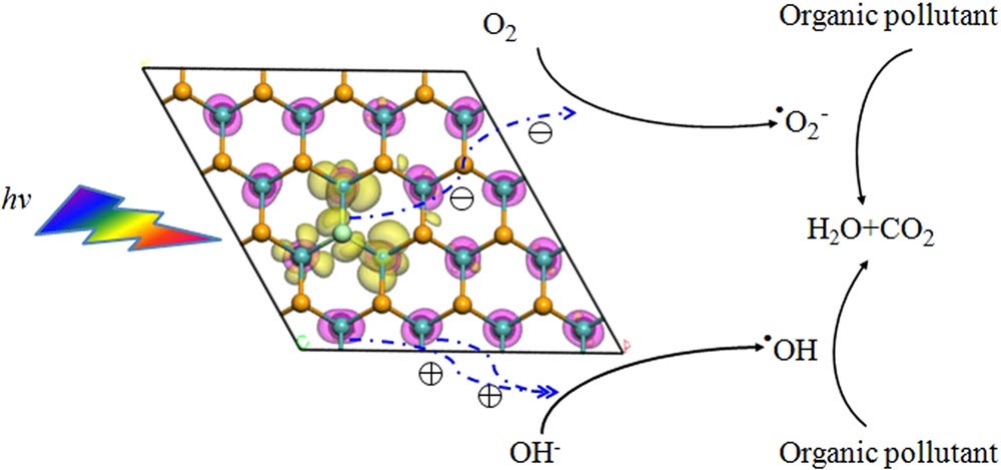
The mechanism of photocatalytic degrading organic pollutant and the charge dynamics in VIIA doped monolayer MoSe_2_. The purple and yellow regions represent HOMO and LUMO, respectively. Under light irradiation, the photogenerated e^−^ moves to Mo atoms and VIIA group atoms in the yellow region, while, photogenerated h^+^ moves only on the Mo atoms in the purple region. This represents the separation of photogenerated e^−^/h^+^ in real space.

O_2_^**−**^ ion and OH radical are important oxidants for the degradation of organic pollutants. As an example, the schematic mechanism of photocatalytic degrading process is shown in Fig. 5 for VIIA (F, Cl, Br and I) group doped MoSe_2_ system. Under light irradiation, the e^−^ and h^+^ will be generated at the yellow and purple region, respectively. Next, O_2_^**−**^ ions and ^·^OH radicals can be reduced or oxidized by the e^−^ or h^+^, simultaneously. And they can oxidize the organic pollutant into H_2_O and CO_2_. Thus, due to the separation of the HOMO and LUMO states in the real space, NM dopants with an odd number of valence electrons doped monolayer MoSe_2_ can suppress the recombination probability of the photogenerated e^−^ and h^+^, and increase the photocatalytic performance.

**Figure 6:**
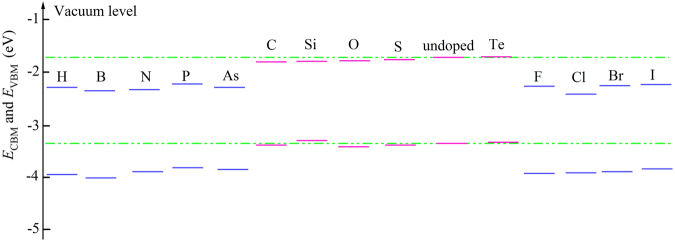
The *E*_CBM_ and *E*_VBM_ of undoped (green dotted line) and NM doped monolayer MoSe_2_. Dopants with odd number of valence electrons doped MoSe_2_ have lower *E*_VBM_, suggesting the oxidation of their photogenerated holes is enhanced compared with undoped MoSe_2_.

Further, the VBM and CBM position are calculated relative to vacuum level^42,43^, see Fig. 6 and Table 1, the oxidation potential of the photogenerated h^+^ is enhanced by 0.44~0.64 eV, while the reduction potential of photogenerated e^−^ is reduces by 0.52~0.69 eV in the NM dopants with odd number of valence electrons (IA, IIIA, VA and VIIA) doped monolayer MoSe_2_. On the contrary, the NM dopants with even number of valence electrons even (IVA and VIA doped MoSe_2_ barely changed the redox potential. Thus, NM dopants with odd number of valence electrons can modify the redox potentials, and tends to generate more ^·^OH radical than O_2_^−^ ion in the photocatalytic reaction. This is consistent with the experimental report that the ^·^OH radical played a dominant role in the visible light photocatalytic degradation of RhB for the B/C-doped TiO_2_^44^.

## Conclusions

In this work, the geometrical structure, electrical transport, redox potentials, molecular state, optical and photocatalytic properties of NM doped monolayer MoSe_2_ are studied by first principle calculations. The *E*_b_ suggests that NM doped MoSe_2_ are energetically favorable compared to Se vacancies, except B- and C-doped MoSe_2_. We have found that NM dopants with odd number of valence electrons doped monolayer MoSe_2_ can be p-type or n-type, while dopants with even valence electrons doped ones tend to be p-type. This provides a useful method to realize bipolar electrical transport properties by tuning the dopants for the applications in optoelectronics and electronics. We have also found NM dopants with odd number of valence electrons doped MoSe_2_ have better photocatalytic performance, due to they can suppress the recombination of photogenerated e^−^ and h^+^, and enhance the oxidation potential of the holes.

## Electronic supplementary material

Supplementary Information
