# Supplementary material for: First-principles study of nonmetal doped monolayer MoSe2 for tunable electronic and photocatalytic properties
Source: Sci Rep. 2017 Dec 6;7:17088. doi: 10.1038/s41598-017-17423-w (PMC5719077; doi:10.1038/s41598-017-17423-w)
Supplement: Supplementary file 1 — Supplementary Information [file 41598_2017_17423_MOESM1_ESM.docx]

**Supporting Information**

First-principles study of nonmetal doped monolayer MoSe_2_ for tunable electronic and photocatalytic properties

Yafei Zhao^a^, Wei Wang^a^, Can Li^b^ and Liang He*^a^

^a^National Laboratory of Solid State Microstructures, School of Electronic Science and Engineering and Collaborative Innovation Center of Advanced Microstructures, Nanjing University, Nanjing 210093, *China*

^b^Center for Coordination Bond Engineering, College of Materials Science and Engineering, China Jiliang University, Hangzhou 310018, China

Corresponding Authors

* L.H: [heliang@nju.edu.cn](mailto:heliang@nju.edu.cn)

**SUPPORTING INFORMATION**

**S1. Optical properties**

The optical properties of all systems have been calculated based on the following equation:$I\left(\omega\right)=\sqrt{2}\omega\left\{\left[\varepsilon_{1}^{2}\left(\omega\right)+i\varepsilon_{2}^{2}\left(\omega\right)\right]^{\frac{1}{2}}-\varepsilon_{1}\left(\omega\right)\right\}^{\frac{1}{2}}$, where the ɛ_1_(ω) and ɛ_2_(ω) are the real part and imaginary part of dielectric function and the related results showed in Fig. S1. It is found that the optical absorption edge of H-, B-, C- and Si-, N- and P-, F-, Cl-, Br- and I-doped MoSe_2_ were red shift less than 43 nm, while the other doped MoSe_2_ were blue shift less than 9 nm. Meanwhile, the optical absorption areas in the range of 300 to 900 nm is increase or reduce less than 8.7% compare with undoped MoSe_2_, due to the impurity levels in the band gap significantly decrease the maximum energy gap.


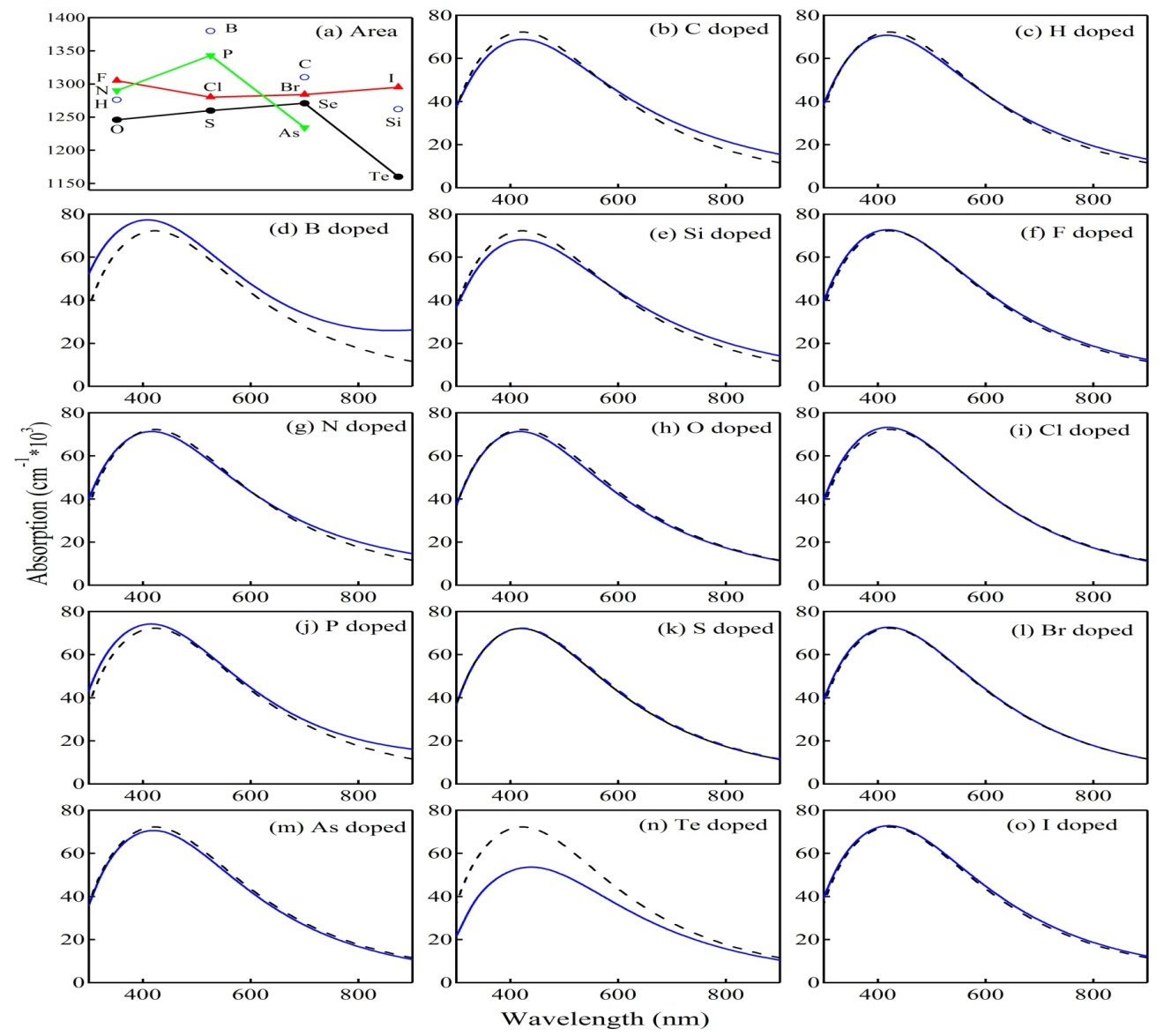


Figure S1. (Color online) (b) - (o) The optical absorption curves and (a) the optical absorption area of undoped and NM doped monolayer MoSe_2_ in the range of 300 to 900 nm. The black dotted (blue solid) line represent the undoped (doped) systems.

**S2.** **The highest occupied molecular orbital (HOMO) and lowest unoccupied molecular orbital (LOMO)**

For undoped MoSe_2_, the HOMO and LUMO were made up of Mo-4*d* states, thus they are located at all the Mo atoms simultaneously (Fig. S2(k)). And the photogenerated electrons (e^-^) and holes (h^+^) will be easily recombined on Mo atoms, which reduce the photocatalytic efficiency. For IVA (C and Si) group doped MoSe_2_, both the HOMO and LUMO mainly locate at the NM atom and its three nearest Mo atoms. For VIA (O, S and Te) group doped MoSe_2_, similar to undoped MoSe_2_, both the HOMO and LUMO mainly locate at the Mo atoms across the film. Thus, in these two cases, the photogenerated electrons e^-^/h^+^ have a good chance to recombine.

For IA (H) group and VIIA (F, Cl, Br and I) group doped MoSe_2_, the distribution of HOMO states is the same as the undoped MoSe_2_, while the LUMO states are mainly located at the NM atom and its three nearest Mo atoms. For IIIA (B) doped MoSe_2_, the distribution is opposite to them. For VA (N, P and As) group doped MoSe_2_, the HOMO states locate at around NM atom and its surrounding Mo atoms, and LUMO states locate at Mo atoms further away from NM atoms. In these three cases, the HOMO and LUMO states are separated in the space, and this can effectively promote the separation of photogenerated e^-^/h^+^, and reduce their recombination probability. Thus, we can conclude that NM dopants with an odd number of valence electrons doped monolayer MoSe_2_ have better photocatalytic performance due to the separation of HOMO and LUMO.


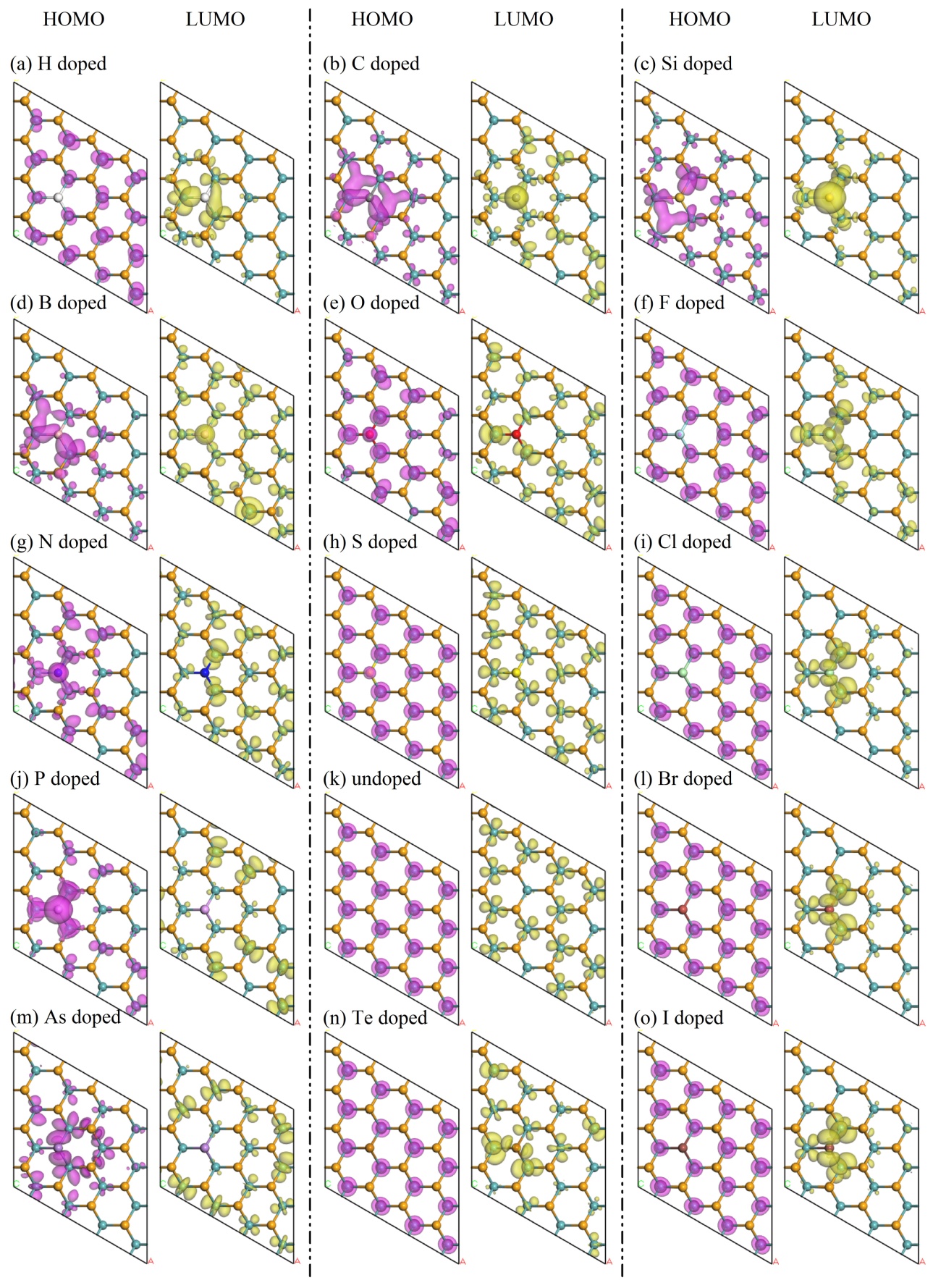


Figure S2. (Color online) HOMO and LUMO of undoped and NM doped monolayer MoSe_2_.
